# Evaluation of Xpert MTB/RIF for the Diagnosis of Lymphatic Tuberculosis

**DOI:** 10.1155/2020/1968487

**Published:** 2020-06-28

**Authors:** Hou-He Li, Zhi-Jian He, Jia-Qi Liang, Gui-Lin Li, Tian-Ao Xie, Ye-Ling Liu, Zhong-Wei Li, Xuan-Cheng Feng, Yong Xia, Xu-Guang Guo

**Affiliations:** ^1^Department of Clinical Medicine, The Third Clinical School of Guangzhou Medical University, Guangzhou 511436, China; ^2^Nanshan School, Guangzhou Medical University, Guangzhou 511436, China; ^3^Department of Clinical Laboratory Medicine, The Third Affiliated Hospital of Guangzhou Medical University, Guangzhou 510150, China; ^4^Key Laboratory for Major Obstetric Diseases of Guangdong Province, The Third Affiliated Hospital of Guangzhou Medical University, Guangzhou 510150, China; ^5^Key Laboratory of Reproduction and Genetics of Guangdong Higher Education Institutes, The Third Affiliated Hospital of Guangzhou Medical University, Guangzhou 510150, China; ^6^Stomatological Hospital of Guangzhou Medical University, 510150, China

## Abstract

**Background:**

The World Health Organization approved the use of Xpert MTB/RIF for the detection of *Mycobacterium tuberculosis* DNA, which has significantly improved the diagnosis of tuberculosis. In this study, our main objective was to evaluate the diagnostic efficacy of Xpert MTB/RIF for lymphoid tuberculosis to determine whether Xpert MTB/RIF could be used as a routine detection method.

**Materials and Methods:**

We searched four databases for the relevant literature published from May 2007 to December 2019. The quality of the literature was evaluated with reference to the evaluation criteria. Data that were extracted from the literature on Xpert MTB/RIF diagnosis of lymphatic tuberculosis were used to plot the summary receiver operating characteristic (SROC) curve, after which the software was used to combine and analyze the accuracy of these data.

**Results:**

A total of 27 studies were included. The sensitivity of Xpert MTB/RIF for detecting lymphatic tuberculosis was 0.79 (95% CI (0.77, 0.81)), the specificity was 0.88 (95% CI (0.87, 0.90)), and the positive likelihood ratio (PLR) was 7.21 (95% CI (4.93, 10.55)). In addition, the negative likelihood ratio (NLR) was 0.25 (95% CI (0.19, 0.32)) and the diagnostic odds ratio (DOR) was 40.23 (95% CI (24.53, 65.98)). At the same time, we used the extracted data to make the SROC curve, obtaining the following parameters: area under the curve (AUC) = 0.9144, *Q* = 0.8470 (SE = 0.0163).

**Conclusion:**

Xpert MTB/RIF has high accuracy in detecting lymphatic tuberculosis, and it can be used to quickly and easily diagnose lymphatic tuberculosis at an early stage as a general method.

## 1. Background

Tuberculosis is the leading infectious cause of death worldwide. It is caused by *M. tuberculosis* that invades the human body, and especially lungs. It can also cause extrapulmonary tuberculosis, which is caused when the bacterium infects the lungs and then spreads through the lymphatic system, invading other organs of the body [1]. Lymphatic tuberculosis is one of the most ordinary kinds of extrapulmonary tuberculosis [2]. In resource-poor countries with a high burden of lymphatic tuberculosis, quick and accurate detection and diagnosis of patients suffering from tuberculosis have become important factors in the control and treatment of lymphatic tuberculosis.

Conventional methods for detecting lymphatic tuberculosis include smear microscopy, culture, or cytology; nevertheless, these methods have certain limitations. Mycobacterial culture can be used as a reference standard but is time-consuming and requires an operator or contact. With a reasonably high level of biosafety, cytological methods for detecting lymphadenopathy require expert human cytological interpretation, while small laboratories often do not have equipment such as fluorescence microscopes or light-emitting diode (LED) microscopes. These factors make it impossible to accurately and quickly diagnose patients with lymphatic tuberculosis in a low resource environment [3].

Xpert MTB/RIF, a complex for detecting *M. tuberculosis*, is a box-based isothermal nucleic acid amplification test, which requires less time to detect *M. tuberculosis*. It is also characterized by short cycle, speed, automaticity, and operator need to have the advantage of too high professional skills [4]. In 2010, the World Health Organization was first to recognize Xpert MTB/RIF for the detection of DNA from *M. tuberculosis* and rapid diagnosis of the function of tuberculosis [5]. According to the predictions and calculations of the World Health Organization, the cost of Xpert is almost the same as that of *M. tuberculosis* culture. However, large-scale implementation of Xpert does not require specialized equipment or culture of a large number of talents to meet the culture process demand. In relation to the tuberculosis control plan, some countries are ready to abandon traditional mycobacterial culture and switch to Xpert for the detection of *M. tuberculosis* [6]. There are few systematic reviews of the accuracy of Xpert MTB/RIF for the detection of lymphatic tuberculosis at home and abroad. Therefore, we conducted a meta-analysis of the sensitivity of Xpert MTB/RIF for the diagnosis of lymphatic tuberculosis by evidence-based medicine.

## 2. Materials and Methods

### 2.1. Overall Process

We first confirmed the inclusion and exclusion criteria and relevant keywords, which was followed by a literature search, preliminary screening, and rescreening. Next, data extraction and literature quality evaluation were performed, after which the statistical analysis was performed; the specific processes are shown in S1.

### 2.2. Inclusion and Exclusion Criteria

The inclusion criteria were the following: (1) subjects: clinical specimens of lymph node tissues identified as tuberculous lymphadenitis by test methods, and tuberculosis detected in the lesions; (2) types of study: analytical test for diagnostic accuracy data for identification of bacterial species could be extracted from literature; only literature in English literature was considered; and (3) diagnostic test method: detection of *M. tuberculosis* using Xpert MTB/RIF.

Exclusion criteria were (1) duplicate literature; (2) conference abstracts, lectures, letters, and reviews; (3) literature lacking reference standards; and (4) literature lacking a fourfold table.

### 2.3. Search Strategy

The “Xpert” and “Lymph” keywords were used to jointly search the PubMed database, the Web of Science database, the Embase database, and the Cochrane Library database for relevant documents published between May 2007 and December 2019. Possible matches were also retrieved from the related references and were incorporated with standard literature.

### 2.4. Selection of Literature Research and Data Extraction and Quality Evaluation

#### 2.4.1. Selection of Research and Extraction of Data

Four evaluators independently searched for the “Xpert” and “Lymph” keywords in view of the predefined inclusion and exclusion criteria. PubMed database, Web of Science database, the Embase database, and the Cochrane Library database were searched for the relevant literature published between May 2007 and May 2019. The studies were extracted and analyzed, and those documents that met the inclusion criteria were evaluated for quality. If the four evaluators cannot reach an agreement on the results of data extraction, analysis, and evaluation results, differences would be resolved by review and negotiation. If differences remained, the third party would be introduced to reach consensus, who was blinded to the authors or institutions of the studies undergoing review. The extracted data included author, year, type of experimental design (prospective or retrospective), gold standard, classification, type of specimens (FNA or tissue), TP, FP, FN, TN, errors and the total number of samples, and other related information, during which a fourfold table was regarded as the standardized data extraction form. In the process of data extraction, we found that the gold standards are culture and composite reference standard (CRS), which even appears in some of the literature at the same time. We regard such articles as two articles for extraction and label “a” with culture as the gold standard and “b” with CRS as the gold standard in the figure (Table 1).

#### 2.4.2. Quality Evaluation of Literature

The literature was evaluated for quality, mainly based on the quality evaluation criteria of the diagnostic test recommended by the Cochrane Collaboration Network-QUADAS-2 [7]. The quality of the included documents was based on 11 evaluation questions in the QUADAS-2 standard. The scoring criteria were “Yes (Y)”, which equaled to 1 point, “No (N)”, which equaled to 0, and “Unclear (UC)”, which equaled to 0.5 points.

## 3. Statistical Analysis

The following data were extracted: total number of samples, specificity, sensitivity, true positive, false positive, true negative, and false negative. The data of Xpert MTB/RIF for the diagnosis of lymphatic tuberculosis were accurately combined and analyzed by Stata 12.0 software and meta-disc v.1.4 software. The accuracy of each diagnostic method was analyzed using a random-effects model and presented in the form of a funnel plot and a forest plot, respectively. We used the form of a forest map to obtain an accurate estimate for each study in a general overview and then analyzed and interpreted the estimates of the forest map summary. In the forest map, a *P* value of <0.05 represented a statistically significant difference. If the results of the data graphs such as the forest map and the funnel map were not satisfactory, the four evaluators solved the problem by negotiating and inspecting whether the effect model (fixed-effects model and random-effects model) and effect quantity (categorical variable and continuous variable) were satisfactory.

## 4. Results

### 4.1. Characteristics of Inclusion Studies

By applying the above-described search strategy, 260 articles were identified. After deleting duplicate articles, 180 articles were left. Next, we screened the title, abstract, and conclusion of the research. Finally, we conducted a full-text review and meta-analysis of 27 articles [3, 8–33] (S1, Table 1). The sample number of each article ranged from 25 to 348.

Among the 27 documents included, 21 were prospective and 6 were retrospective studies. Besides, in these 27 articles, the sample types were fine needle aspiration (FNA) and tissue, and the storage conditions were also divided into frozen and fresh. In this article, we only present part of the type of specimen.

### 4.2. Threshold Effect Analysis

**Figure 1:**
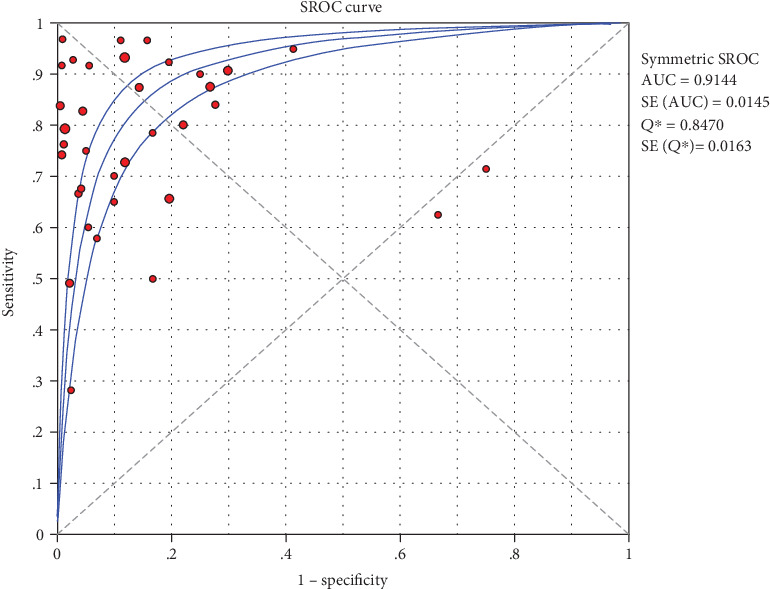
The SROC curve for the combined sensitivity of Xpert MTB/RIF.

As can be seen from the SROC curve (Figure 1), it did not have a “shoulder arm” distribution. At the same time, due to the threshold effect analysis, Spearman correlation coefficient was 0.100, and the *P* value was 0.182 (*P* > 0.05), which suggested that the included articles did not have the threshold effect.

### 4.3. Heterogeneity Analysis of Nonthreshold Effect

A forest map, which was used to plot the ratio, revealed that there was no heterogeneity in nonthreshold effects: Cochran‐*Q* = 99.82, *P* ≤ 0.01 (*P* < 0.05), inconsistency = 65.9% (inconsistent > 50%).

### 4.4. SROC Curve

The obtained meta-analysis data were used to generate the SROC curve, which illustrated the total score of the test (Figure 1). As the chart shows, AUC was 0.9144, and *Q* index was 0.8470 (SE = 0.0163). Since AUC and *Q* index was close to 1, this indicated a high overall accuracy, which suggests that Xpert MTB/RIF can be used to diagnose lymphatic tuberculosis more accurately.

### 4.5. Merge Analysis Results

**Figure 2:**
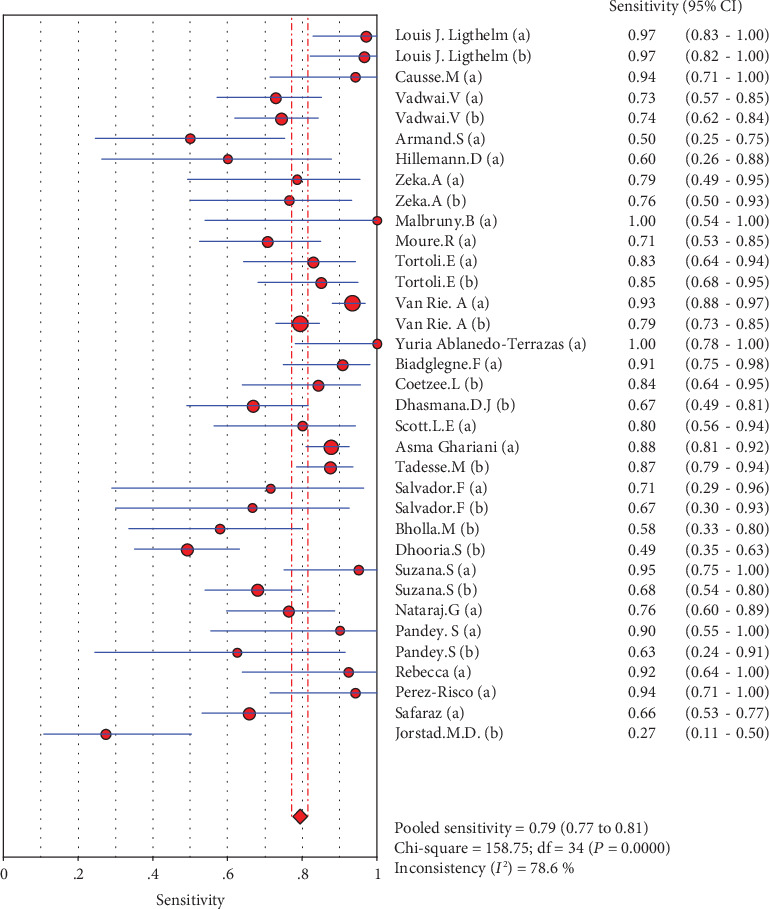
Forest plots for the combined sensitivity of Xpert MTB/RIF.

**Figure 3:**
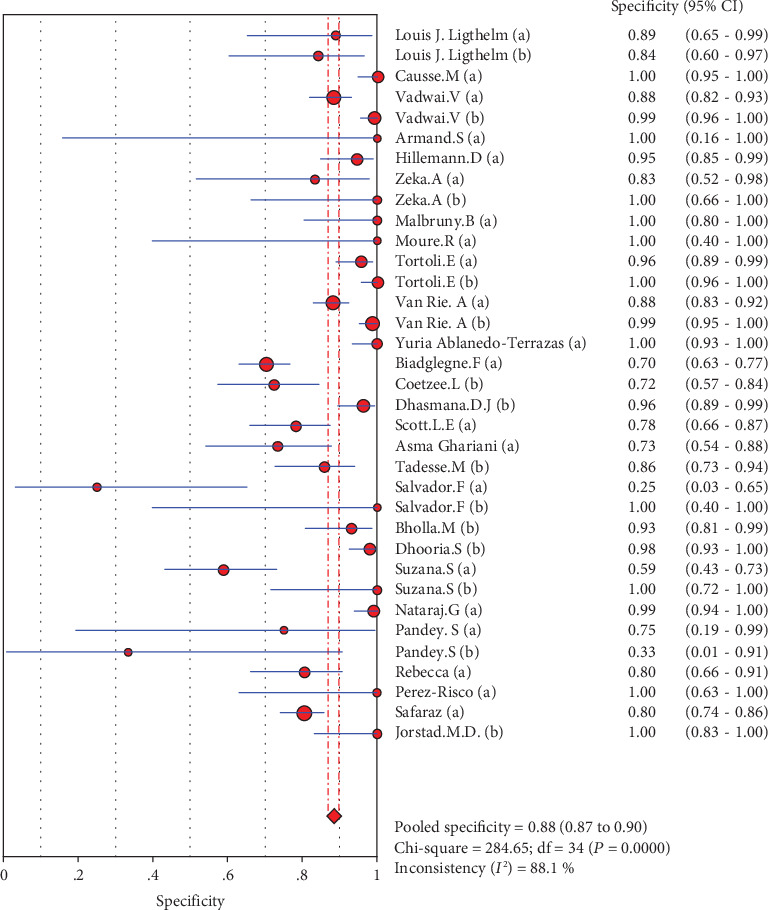
Forest plots for the combined specificity of Xpert MTB/RIF.

**Figure 4:**
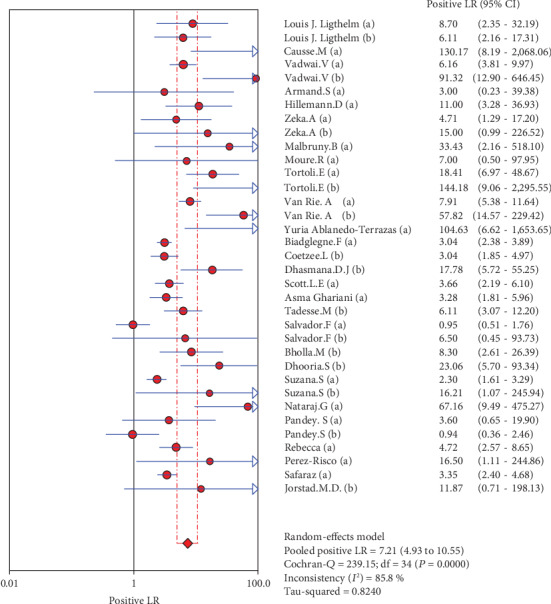
Forest plots for the combined positive LR of Xpert MTB/RIF.

**Figure 5:**
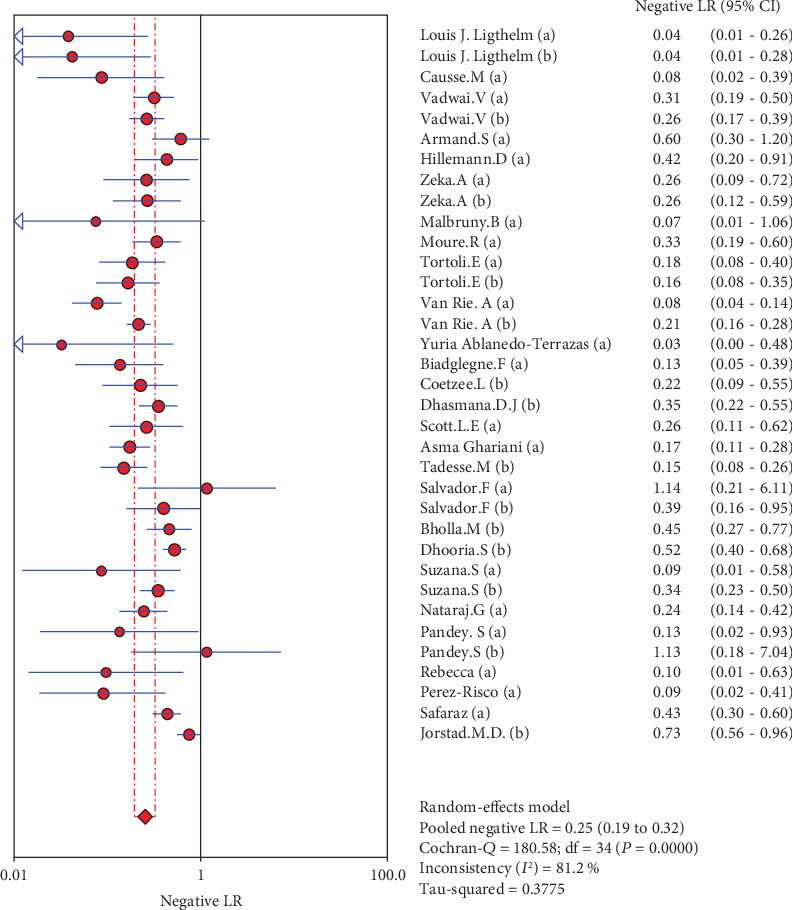
Forest plots for the combined negative LR of Xpert MTB/RIF.

**Figure 6:**
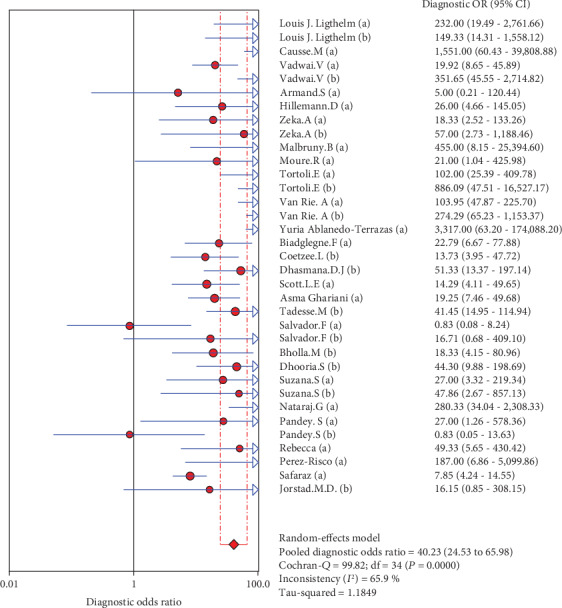
Forest plots for the combined diagnostic OR of Xpert MTB/RIF.

The results are shown in Figures 2–6. The sensitivity of Xpert MTB/RIF in the diagnosis of lymph node nuclei was 0.79 (95% CI (0.77, 0.81)), the specificity was 0.88 (95% CI (0.87, 0.90), the positive LR was 7.21 (95% CI (4.93, 10.55)), and the negative LR was 0.25 (95% CI (0.19, 0.32)). The combined diagnostic ratio was 40.23 (95% CI (24.53, 65.98); Figure 6).

### 4.6. Publication Bias

**Figure 7:**
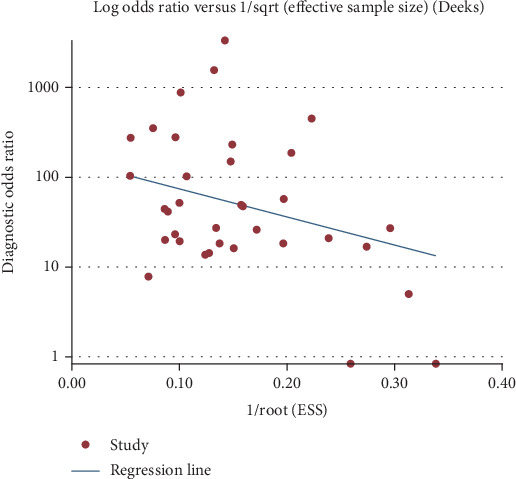
Deeks funnel asymmetrical test evaluates publication bias in Xpert MTB/RIF diagnostic odds ratio estimates for lymphoid tuberculosis.

In order to explore whether there was publication bias, we used Deeks funnel graph asymmetry to test our data. Despite the asymmetry shown in Figure 1, the results of this funnel diagram need to be studied due to the limited number of the included studies. However, the Egger test showed that *P* > 0.05 was not statistically significant, which confirmed that there was no bias in this study (Figure 7).

## 5. Discussion

In the current study, we included 27 studies and extracted 33 fourfold figures to make a systematic diagnosis of lymphatic tuberculosis by Xpert MTB/RIF. The results of the evaluation showed that Xpert MTB/RIF had a sensitivity of 0.79 (95% CI (0.77, 0.81)), a specificity of 0.88 (95% CI (0.87, 0.90)), a PLR of 7.21 (95% CI (4.93, 10.55)), a NLR of 0.25 (95% CI (0.19, 0.32)), and a DOR of 40.23 (95% CI (24.53, 65.98)). At the same time, we also used the extracted data to make the SROC curve and obtained the following parameters: AUC of 0.9144 and *Q* of 0.8470 (SE = 0.0163). The AUC was close to 1, which suggests a relatively high overall accuracy of the study. Taken together, the Xpert MTB/RIF technique has a better diagnostic effect for lymphatic tuberculosis.

Currently, *M. tuberculosis* culture is widely used as the gold standard for the diagnosis of tuberculosis. However, this technique has the disadvantage of being time-consuming since it can take up to several weeks to confirm the results [34, 35]. This approach also requires a cumbersome experimental design, which leads to a lower detection rate, thus making it a less preferable technique for early diagnosis of *M. tuberculosis*. In contrast, Xpert MTB/RIF technology provides fast diagnostic results in less than 2 hours, which significantly increases the clinical efficiency of tuberculosis diagnosis. At the same time, Xpert MTB/RIF is more porFigure and widely used than *M. tuberculosis* culture, which is difficult to perform in low-income countries. Its automation features also reduce the requirements for medical personnel [36]. The World Health Organization (WHO) approved this technology in December 2010, which has been considered the major advancement in global tuberculosis control. The WHO believes it has the potential to revolutionize and change the treatment and control of tuberculosis [5]. Besides, in 2013, the WHO updated the policy to detect MTB from cerebrospinal fluid, lymph nodes, and other inflammatory cases by using the GeneXpert MTB/RIF test [37]. Because of this, Xpert MTB/RIF is one of the preferred options for the diagnosis of lymphatic tuberculosis.

Since Xpert MTB/RIF is prone to “error” in clinical application, that is, it cannot be detected, we also extracted the number of “error” in various studies during data extraction (Table 1). Of the 27 studies we included, most recorded the number of errors in lymph detection. The number of errors in most studies is 0. Only two studies, although illustrating the existence of the “error” situation, did not record the number of errors in lymph detection [14, 29]. In Gidado et al.'s research [38], we learned that Xpert MTB/RIF detection has five types of error causes: Type A: failure caused by unsuitable temperature, including dust accumulation and fan failure; Type B: failure caused by human factors, including unqualified specimens and excessive filling; Type C: kit failure, mostly due to improper storage of the kit; Type D: failure caused by poor line connection, power supply fluctuations, etc.; and Type E: machine failure caused by component failure or module failure.

The results show that the “error” situation often occurs in the detection of extrapulmonary samples. Some researchers pointed out that this situation may be due to the presence of PCR inhibitors in the extrapulmonary samples, which prevented the test from being performed [15]. In another study, the researchers believed that the sample reagent buffer used in the Xpert analysis was originally designed for liquefaction of mucus-like sputum specimens, which may not be suitable for a variety of extrapulmonary specimens, resulting in detection errors [33]. Obviously, for extrapulmonary samples, the most common type of error is B. Operators should follow protocol and national standard operating procedures (SOPs) strictly to perform operations to minimize the occurrence of errors [38].

During the analysis of the extracted data, we found that the Xpert MTB/RIF test results and culture as the gold standard test results are very different in some studies. After reviewing the 27 articles included in the analysis, we summarized some of the main reasons. First, the culture method needs to use the N-acetyl-L-cysteine and sodium hydroxide method (NALC/NaOH) for digestion and purification of the sample, which may result in the loss of live bacteria and uneven distribution of bacteria in the sample, thus making the culture test result negative [13, 14, 23]. Second, FNA is used as a sample by many studies. If the needle used for aspiration is not rinsed clearly, it will lead to an increase in the positive rate of culture testing [3]. If the tuberculosis is purulent, a similar situation will occur [3, 12]. Third, Xpert MTB/RIF obtains the detection results by detecting the rpoB gene in *M. tuberculosis*. If *M. tuberculosis* in the sample has rpoB gene defects, it cannot be detected, which will increase the negative rate of Xpert MTB/RIF testing [33]. These circumstances may cause the detection results of the two methods to be very different.

During this study, a related study was published [39], whose author is Yu et al. Compared with Yu et al.'s study, all the literature included in our study were from four English databases, which ensured higher data credibility. At the same time, Yu et al.'s study was analyzed separately according to the types of extracts, while we combined the data directly, which may be affected by the type of samples, but also more intuitively reflect the diagnostic efficacy of Xpert MTB/RIF technology for lymphatic tuberculosis. This is also one of the strengths of our article.

Our study also has some limitations. First of all, we only extracted data from the published literature in the four English databases, which may lead to defects in the comprehensiveness of this study. Secondly, although some of the studies [19, 22, 31–33] adhere to the principle of rigor, a large number of studies fail to repeat the tests on the specimens at least one time due to various objective factors. Lack of repeated tests may lead to inaccurate final analysis results.

In summary, Xpert MTB/RIF is a highly sensitive and highly specific method for diagnosing lymphatic tuberculosis, which is of great value for the early diagnosis of lymphatic tuberculosis. It can reduce treatment time and the use of antibiotics, reduce the suffering of patients, and save their lives. In the future, this technology could become the optimal auxiliary diagnostic method for tuberculosis.

## 6. Conclusions

In conclusion, Xpert MTB/RIF is highly sensitive and specific for the diagnosis of lymphoid tuberculosis, and it also has the advantages of convenience, simple and fast operation, and a good economy. Xpert MTB/RIF can be used as a routine detection method for lymphoid tuberculosis in the early clinical stage.

## Figures and Tables

**Table 1:** Characteristics of the included studies.

Author	Year	Design	Gold standard	Results				Type of specimens	Error
				TP	FP	FN	TN		
Ligthelm et al. (a) [11]	2011	Retrospective	Culture	29	2	1	16	FNA	0
Ligthelm et al. (b) [11]	2011	Retrospective	CRS	28	3	1	16	FNA	0
Causse et al. (a) [16]	2011	Prospective	Culture	16	0	1	70	Tissue	0
Vadwai et al. (a) [32]	2011	Prospective	Culture	32	17	12	127	Tissue	0
Vadwai et al. (b) [32]	2011	Prospective	CRS	49	1	17	122	Tissue	0
Armand et al. (a) [15]	2011	Retrospective	Culture	8	0	8	2	Tissue	2
Hillemann et al. (a) [29]	2011	Prospective	Culture	6	3	4	52	Tissue	No data
Zeka et al. (a) [22]	2011	Retrospective	Culture	11	2	3	10	Tissue	0
Zeka et al. (b) [22]	2011	Retrospective	CRS	13	0	4	9	Tissue	0
Malbruny et al. (a) [27]	2011	Prospective	Culture	6	0	0	17	FNA	0
Moure et al. (a) [20]	2012	Retrospective	Culture	24	0	10	4	Tissue	0
Tortoli et al. (a) [14]	2012	Retrospective	Culture	24	4	5	85	Tissue	No data
Tortoli et al. (b) [14]	2012	Retrospective	CRS	28	0	5	85	Tissue	No data
Van Rie et al. (a) [17]	2013	Prospective	Culture	139	23	10	172	FNA	5
Van Rie et al. (b) [17]	2013	Prospective	CRS	160	2	42	144	FNA	5
Ablanedo-Terrazas et al. (a) [12]	2014	Prospective	Culture	15	0	0	53	FNA	0
Biadglegne et al. (a) [19]	2014	Prospective	Culture	29	56	3	132	FNA	7
Coetzee et al. (b) [28]	2014	Prospective	CRS	21	13	4	34	FNA	1
Dhasmana et al. (b) [26]	2014	Prospective	CRS	24	3	12	77	FNA	0
Scott et al. (a) [18]	2014	Prospective	Culture	16	14	4	50	FNA	0
Ghariani et al. (a) [13]	2015	Prospective	Culture	126	8	18	22	Tissue	0
Tadesse et al. (b) [24]	2015	Prospective	CRS	76	7	11	42	FNA	2
Salvador et al. (b) [21]	2015	Prospective	Culture	6	0	3	4	Tissue	0
Salvador et al. (a) [21]	2015	Prospective	Culture	5	6	2	2	FNA	0
Bholla et al. (b) [3]	2016	Prospective	CRS	11	3	8	40	FNA	0
Dhooria et al. (b) [30]	2016	Prospective	CRS	26	2	27	92	FNA	0
Suzana et al. (a) [31]	2016	Prospective	Culture	19	19	1	27	Tissue	0
Suzana et al. (b) [31]	2016	Prospective	CRS	38	0	18	11	Tissue	0
Nataraj et al. (a) [33]	2016	Prospective	Culture	29	1	9	87	FNA	0
Pandey et al. (b) [23]	2016	Prospective	Culture	5	2	3	1	Tissue	0
Pandey et al. (a) [23]	2016	Prospective	Culture	9	1	1	3	FNA	0
Rebecca et al. (a) [8]	2018	Retrospective	Culture	12	9	1	37	Tissue	0
Perez-Risco et al. (a) [10]	2018	Prospective	Culture	16	0	1	8	FNA	0
Sarfaraz et al. (a) [9]	2018	Prospective	Culture	44	38	23	156	FNA	0
Jørstad et al. (b) [25]	2018	Prospective	CRS	6	0	16	20	FNA	0

## Data Availability

All data generated or analyzed during this study are included in this published article and its supplementary information files.

## Abbreviations

*M. tuberculosis*: *Mycobacterium tuberculosis*
CRS: Composite reference standard
FNA: Fine needle aspiration
WHO: World Health Organization.

## Supplementary Materials

Supplementary Materials S1 flow diagram of study identification and inclusion.
