# Supplementary material for: Evaluation of Xpert MTB/RIF for the Diagnosis of Lymphatic Tuberculosis
Source: Biomed Res Int. 2020 Jun 28;2020:1968487. doi: 10.1155/2020/1968487 (PMC7338981; doi:10.1155/2020/1968487)
Supplement: Supplementary Materials — S1 flow diagram of study identification and inclusion. [file 1968487.f1.pdf]

PRISMA 2009 Flow Diagram

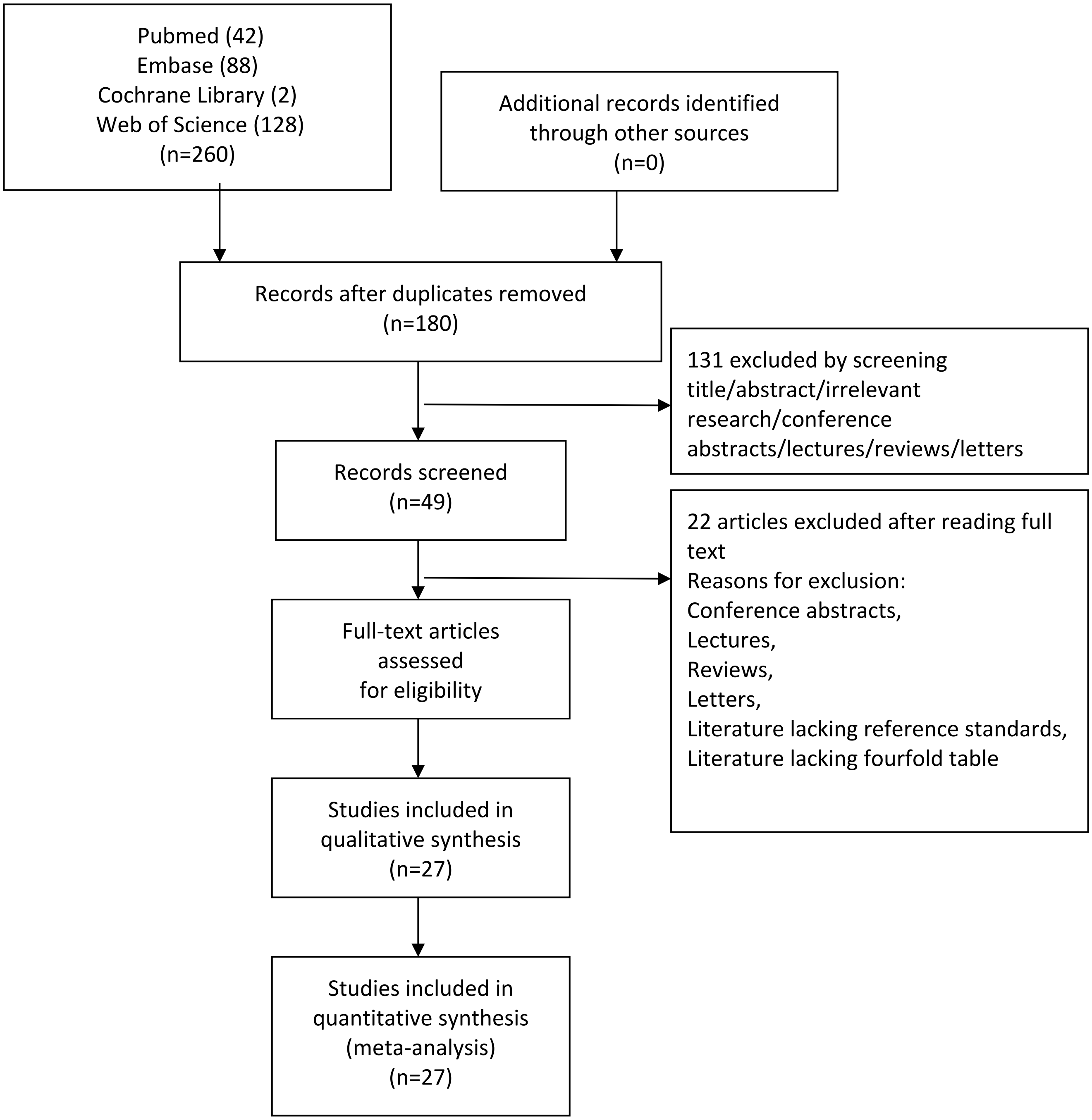

From: Moher D, Liberati A, Tetzlaff J, Altman DG, The PRISMA Group (2009). *P*referred *R*eporting *I*tems for *S*ystematic Reviews and *M*eta-*A*nalyses: The PRISMA Statement. PLoS Med 6(6): e1000097. doi:10.1371/journal.pmed1000097

For more information, visit www.prisma-statement.org.
